# Recurrent immunosuppressive-responsive myocarditis in a patient with desmoplakin cardiomyopathy: a case report

**DOI:** 10.1093/ehjcr/ytae129

**Published:** 2024-03-13

**Authors:** Hayden McColl, Rachael Cordina, Sean Lal, Matthew Parker, Imre Hunyor, Caroline Medi, Belinda Gray

**Affiliations:** Department of Cardiology, Royal Prince Alfred Hospital, 50 Missenden Road, Camperdown, New South Wales 2050, Australia; Faculty of Medicine and Health, The University of Sydney, Science Road, Camperdown, New South Wales 2050, Australia; Department of Cardiology, Royal Prince Alfred Hospital, 50 Missenden Road, Camperdown, New South Wales 2050, Australia; Faculty of Medicine and Health, The University of Sydney, Science Road, Camperdown, New South Wales 2050, Australia; Department of Cardiology, Royal Prince Alfred Hospital, 50 Missenden Road, Camperdown, New South Wales 2050, Australia; Faculty of Medicine and Health, The University of Sydney, Science Road, Camperdown, New South Wales 2050, Australia; Department of Cardiology, Royal Prince Alfred Hospital, 50 Missenden Road, Camperdown, New South Wales 2050, Australia; Faculty of Medicine and Health, The University of Sydney, Science Road, Camperdown, New South Wales 2050, Australia; Department of Cardiology, Royal Prince Alfred Hospital, 50 Missenden Road, Camperdown, New South Wales 2050, Australia; Faculty of Medicine and Health, The University of Sydney, Science Road, Camperdown, New South Wales 2050, Australia; Department of Cardiology, Royal Prince Alfred Hospital, 50 Missenden Road, Camperdown, New South Wales 2050, Australia; Faculty of Medicine and Health, The University of Sydney, Science Road, Camperdown, New South Wales 2050, Australia; Department of Cardiology, Royal Prince Alfred Hospital, 50 Missenden Road, Camperdown, New South Wales 2050, Australia; Faculty of Medicine and Health, The University of Sydney, Science Road, Camperdown, New South Wales 2050, Australia

**Keywords:** Arrhythmogenic cardiomyopathy, Genetic cardiomyopathy, Cardiac MRI, Desmoplakin cardiomyopathy, Case report

## Abstract

**Background:**

Desmoplakin (DSP) cardiomyopathy is a rare genetic condition characterized by repeated inflammatory myocardial injury and is associated with ventricular arrhythmia and sudden cardiac death. Diagnosis is challenging and requires a combination of genetic testing and advanced imaging techniques.

**Case summary:**

We present the case of a 38-year-old woman with recurrent episodes of subclinical myocarditis. Investigation using cardiac magnetic resonance imaging (cMRI) and genetic testing revealed a diagnosis of DSP cardiomyopathy. Her disease was initially responsive to corticosteroid therapy but quickly relapsed when treatment was tapered. Management of her condition required significant immunosuppression and the subsequent insertion of an implantable cardiac defibrillator due to her risk of sudden cardiac death.

**Discussion:**

Cardiac MRI and genetic testing are key diagnostic techniques in the assessment of patients with recurrent myocarditis and cardiomyopathy. The management of cardiomyopathies with an inflammatory component is not completely understood; however, there is likely a key role for immune suppression therapies. Furthermore, there are several cardiomyopathy genetic variants including DSP which require careful risk stratification due to an increased risk of sudden cardiac death.

**Learning points**

- Genetic testing should be considered in patients with unexplained cardiomyopathy or recurrent myocarditis.
- Several genetic variants including desmoplakin cardiomyopathy have high rates of ventricular arrhythmia, and implantable cardiac defibrillator insertion may be considered based on genotype alone.
- In patients with recurrent myocarditis secondary to an inflammatory cardiomyopathy, significant immunosuppressive therapy may be required to achieve disease remission and prevent progression.

## Primary specialties involved other than cardiology

Medical genetics, rheumatology, immunology, radiology

## Introduction

Desmoplakin (*DSP*) cardiomyopathy is a rare genetic condition typically leading to a phenotype of a left ventricle (LV) dominant arrhythmogenic cardiomyopathy (ACM).^1,2^ Although the pathogenesis is incompletely understood, one hypothesis postulates repetitive inflammatory myocardial injury leading to fibrosis.^2^ The diagnosis of *DSP* cardiomyopathy typically relies on a combination of cardiac magnetic resonance imaging (MRI) (cMRI) and genetic testing.^3,4^ Establishing the diagnosis of *DSP* cardiomyopathy is particularly important due to its risk of severe ventricular arrhythmias, which may warrant prophylactic insertion of an implantable cardiac defibrillator on the basis of genotype alone. In patients with DSP-mediated ACM, a left ventricular ejection fraction (LVEF) < 55%, ventricular ectopy, and evidence of fibrosis on cMRI have been shown to be associated with an increased risk of severe ventricular arrhythmias.^2^

## Summary figure

**Figure:**
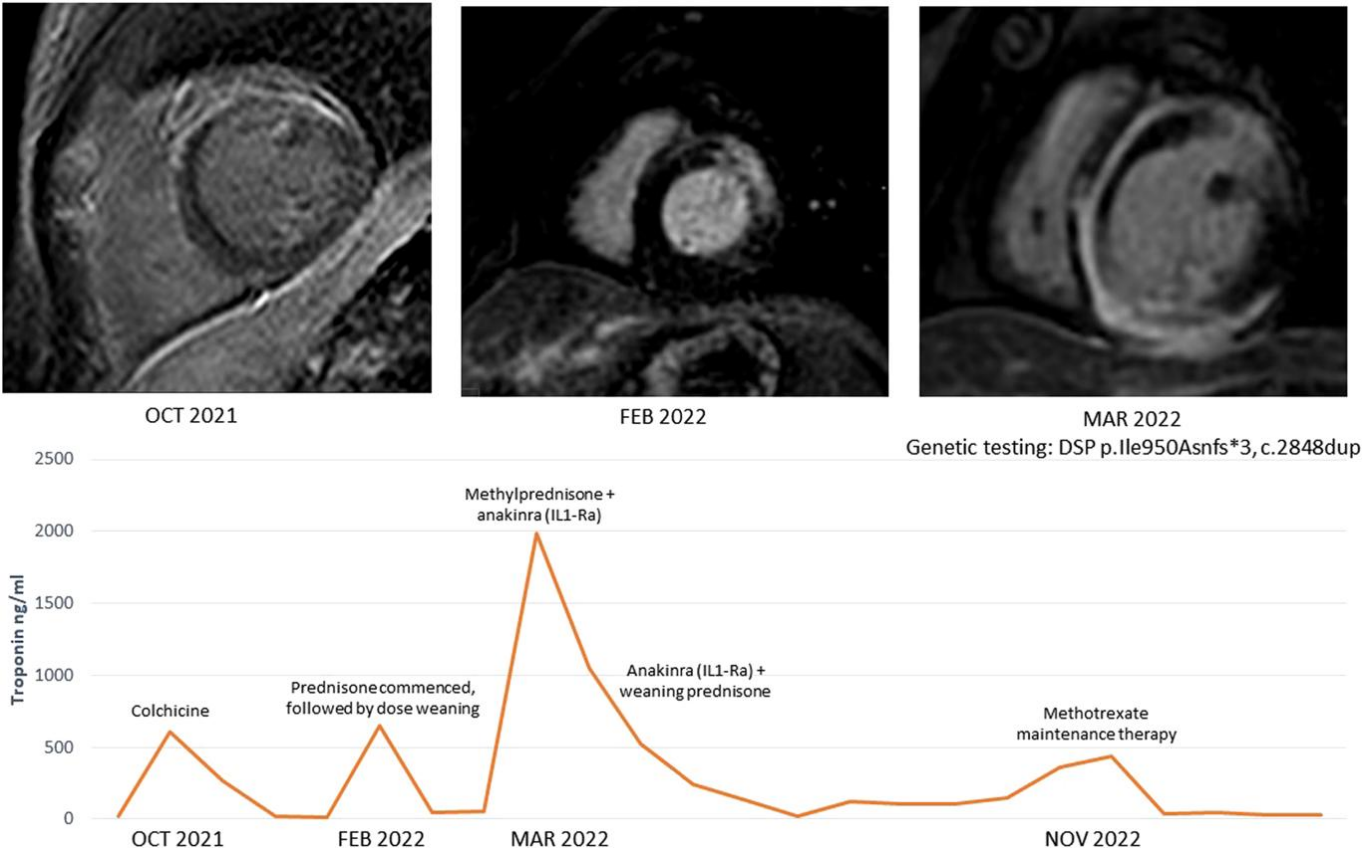


## Case presentation

**Figure 1:**
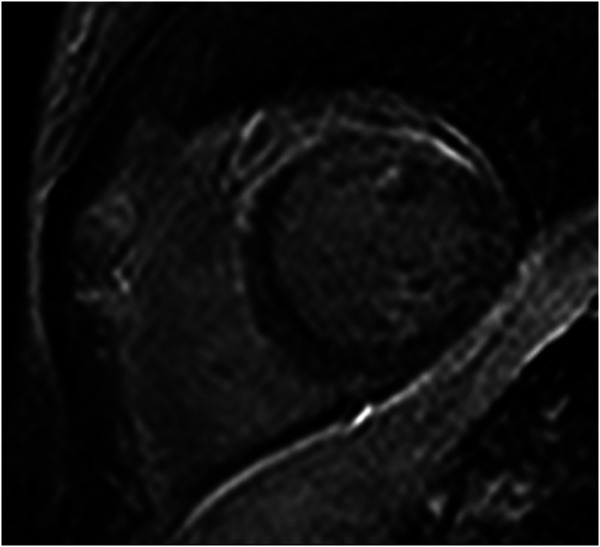
Initial cardiac magnetic resonance imaging demonstrating subepicardial delayed enhancement primarily affecting the anterior myocardium.

**Figure 2:**
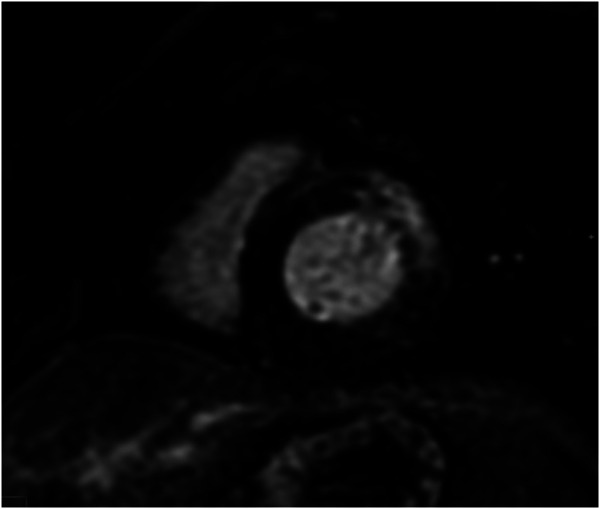
Serial cardiac magnetic resonance imaging demonstrating extensive subepicardial delayed enhancement primarily affecting the anterolateral myocardium.

A 38-year-old woman was transferred from a rural hospital to our centre for further investigation and management of recurrent myocarditis. Her initial diagnosis of myocarditis had been established 11 months prior, in March 2021, after presenting to a district hospital with intermittent paroxysms of sharp non-exertional chest pain and a mildly elevated troponin level (20 ng/L). She was haemodynamically stable with unremarkable physical examination. Of note, she did not have any palmoplantar keratoderma or ‘woolly’ hair. There was no significant past medical history, and there was no family history of sudden cardiac death or cardiomyopathy. Further investigations including 12-lead electrocardiogram (ECG) and computed tomography (CT) pulmonary angiogram were all normal. A transthoracic echocardiogram was performed which demonstrated normal biventricular size and function without evidence of valvular disease or a significant pericardial effusion. A CT coronary angiogram demonstrated no evidence of coronary artery disease and a calcium score of zero. She was commenced on colchicine (500 mcg BD) for management of presumed mild idiopathic myopericarditis. In the 11 months since her initial presentation, she had represented with several flares of myocarditis with significant troponin elevations (>500 ng/L). These flares were commonly asymptomatic, detected on surveillance troponin testing completed during routine follow-up. A cMRI completed in October 2021 revealed mildly increased signal on T2-weighted imaging, together with corresponding subepicardial delayed enhancement of the anterior myocardium which was judged at the time to be consistent with myocarditis (*Figure 1*). Ventricular systolic function was normal with normal wall thickness. Due to the relapsing–remitting pattern to her disease, she was commenced on prednisone 50 mg daily and transferred to our centre for further investigation. A repeat cMRI (February 2022) demonstrated subtle improvement in the previously noted anterior delayed enhancement; however, note was made of extensive near transmural delayed enhancement of the anterior–lateral myocardium with mild associated hypokinesis but without evidence of active inflammation on T2-weighted imaging (*Figure 2*), suggestive of a previous inflammatory/fibrotic process without evidence of active disease.

Screening tests for an underlying systemic causes including autoimmune or connective tissue diseases including ANA, ENA, dsDNA, RF, anti-CCP, ANCA, and serum ACE level were negative. Similarly, there was no evidence of viral or other infective causes of the patient’s symptoms. Positron emission tomography (PET)-CT demonstrated no active myocardial or extracardiac inflammation and specifically no evidence to suggest active sarcoidosis. Myocardial biopsy was considered but not pursued at this time as believed to be low yield due to the lack of active inflammation. Given the recurrent nature of her myocarditis as represented by her significant troponin elevation, immune suppression with azathioprine 50 mg daily was commenced, with weaning prednisone at time of discharge.

**Figure 3:**
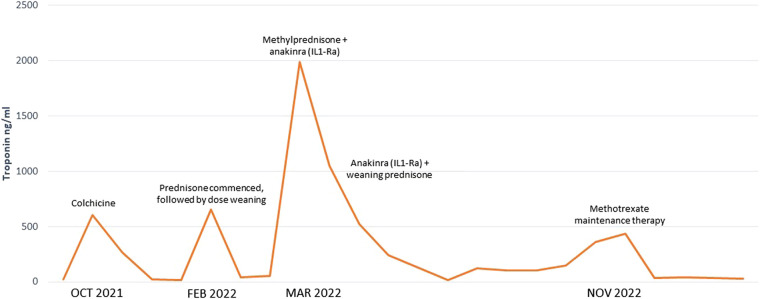
Timeline of the patient’s disease flares indicated by troponin elevation and medical therapy.

Despite combined azathioprine and colchicine, the patient was readmitted with a further asymptomatic flare of myocarditis when the prednisone was weaned to 40 mg daily with a recurrent elevation in troponin (1984 ng/L). Given the refractory nature of her recurrent myocarditis, the decision was made to trial an interleukin-1 receptor antagonist (anakinra 100 mg via sci daily). A single dose of IV methylprednisolone (1000 mg) was also administered. A timeline of the patients’ therapies and flares of disease is presented in *Figure 3*.

**Figure 4:**
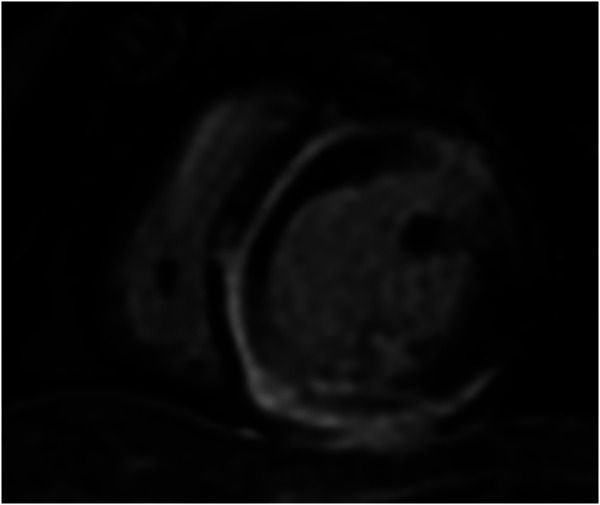
Serial cardiac magnetic resonance imaging demonstrating significant progression of disease with near circumferential subepicardial delayed enhancement of the left ventricular myocardium.

Repeat cMRI 1 month later showed significant progression of late gadolinium enhancement (LGE) with extensive near circumferential ring-like subepicardial delayed enhancement of the LV myocardium (*Figure 4*) with increased myocardial signal on T2-weighted imaging indicative of active inflammation. Left ventricular systolic function was at the lower limits of normal, with normal chamber volumes. The combination of MRI findings on this study was recognized to be consistent with a genetically mediated ACM suspicious for *DSP*. Endomyocardial biopsy was performed at this time; however, the sampled region was normal histologically. During this admission, cardiac telemetry detected short runs of non-sustained ventricular tachycardia (NSVT). An electrophysiology study was negative for inducible VT. A loop recorder was implanted to monitor for further arrhythmia. Cardiac genetic testing was performed and confirmed the presence of a likely pathogenic truncating variant in the *DSP* gene (DSP p.Ile950Asnfs*3, c.2848dup) located within G1, constitutive non-sense–mediated mRNA decay (NMD)-competent region. With the intention of preventing further inflammatory myocardial damage, the patient was commenced on oral methotrexate as a long-term immunosuppressant therapy.

At follow-up 3 months post-discharge, the patient was noted to have multiple runs of asymptomatic NSVT on interrogation of her loop recorder. Given the genetic diagnosis, the high-risk features on CMR, and documented NSVT, the patient underwent insertion of a primary prevention ICD for *DSP*-mediated ACM.

## Discussion

*DSP*-mediated ACM is a rare genetic cardiomyopathy due to pathogenic mutations in *DSP*, the gene encoding DSP, a structural protein responsible for desmosomal binding and the transmission of myocardial forces.^5,6^ The pathogenesis of *DSP* cardiomyopathy is not completely understood; however, DSP variants have been recognized as a unique condition characterized by repeated inflammatory myocardial injury with resultant progressive ventricular fibrosis.^2,3^ The sequelae of these insults include LV or biventricular systolic impairment and the development of a pro-arrhythmic myocardial substrate with a high risk of ventricular arrhythmias and sudden cardiac death. Unlike classical arrhythmogenic right ventricular cardiomyopathy (ARVC), DSP-mediated ACM primarily affects the LV, seen in as many as 79% of cases, and is believed to have a female predominance.^7^ Diagnosis of *DSP*-mediated ACM typically relies on identification of typical MRI findings in association with pathogenic or likely pathogenic *DSP* variants. As demonstrated in the most recent cMRI in our patient (*Figure 4*), the characteristic MRI finding is ring-like epicardial LV fibrosis (can overlap with regions of inflammation) although mid-myocardial delayed enhancement has also been reported.^2,4^ As illustrated in our case, the typical ring-like LGE pattern may not be present at the time of initial assessment which contributes to diagnostic delay in this disease. Ring-like epicardial LGE is not exclusive to *DSP* cardiomyopathy and can additionally be seen in other ACM phenotypes including filamin-C (FLNC), phospholamban, and desmin variants.^8^ As a recently recognized disease, the genetic variants associated with disease as well as the phenotype associated with pathogenic DSP variants may broaden over time.

*DSP*-mediated cardiomyopathy is characterized by diverse clinical presentations including intermittent non-exertional chest pain, palpitations, and/or other symptoms of arrhythmia. Ventricular arrhythmia has been reported in up to 33% of patients.^2,7^ *DSP* cardiomyopathy is often diagnosed when patients present with episodic chest pain reflective of underlying myocardial inflammation during a described ‘hot phase’. As demonstrated in this case, recurrent episodes of inflammation may be asymptomatic and self-limiting, making diagnosis challenging, and is commonly misdiagnosed as myocarditis, highlighting the importance of genetic testing.^9^

Although the association of myocarditis manifesting as a ‘hot phase’ of ACM is well-described, the optimal management of relapsing–remitting myocarditis in the setting of *DSP*-mediated ACM is not well established. If impaired LVEF is present, then patients are commonly managed as per standard heart failure with reduced ejection fraction guidelines. Evidence regarding the management of recurrent inflammation is limited, and decision-making is often based on studies of non-*DSP* inflammatory cardiomyopathies. Treatment often consists of corticosteroids in combination with other immune suppressants including methotrexate, mycophenolate, or more recently IL-1 inhibition.^10^ Our patient demonstrated advanced LGE on CMR prior to commencement of non–corticosteroid-based immunosuppression, so it is unclear if earlier institution of this may have impacted disease progression. Currently outcome specific data for treatments in DSP cardiomyopathy is lacking. As outlined in the 2023 European Society of Cardiology guidelines for the management of cardiomyopathies, patients with *DSP*-mediated ACM are at elevated risk of significant ventricular arrhythmia and sudden cardiac death. Risk factors for this have not been well established; however, there is evidence that burden of LGE, presence of LV impairment, and frequent PVCs may be poor prognostic markers.^1^ This case highlights the importance of genetic testing as several genetic variants including DSP, lamin A/C (LMNA), and FLNC may warrant the insertion of a primary prevention ICD based on genotype alone^3^ Furthermore, in patients with DSP cardiomyopathy, the location of the genetic variant may be an additional risk factor for ventricular arrhythmia in particular truncating variants in the constitutive NMD-competent region as seen in our case.^7^ Additional research is required to allow clinicians to apply genetic testing and sub-typing to individual patient decision-making.

## Conclusion

*DSP* cardiomyopathy is a rare condition characterized by repeated inflammatory myocardial injury, resulting in gradual LV impairment and the development of a pro-arrhythmic substrate. As demonstrated in this case, the clinical presentation can often be subtle with episodes of subacute or asymptomatic myocarditis with progressive myocardial fibrosis. Patients may demonstrate suboptimal response to typical immunosuppressive therapies for myocarditis, and the investigation and management of this disease often requires a multidisciplinary approach. Patients with refractory or recurrent myocarditis should undergo cMRI with consideration for genetic testing. As highlighted in the recent ESC guidelines, genetic testing is of particular importance for the identification of pathogenic genetic variants which may indicate a high-risk phenotype for life-threatening ventricular arrhythmia and may warrant insertion of a primary prevention ICD.

## Data Availability

The data underlying this case report can be made available upon reasonable request to the corresponding author.

## Lead author biography

Hayden McColl is a Cardiology Advanced Trainee at Royal Prince Alfred Hospital and a PhD candidate at the University of Sydney. He has an interest in heart failure and cardiovascular imaging having recently completed a fellowship in cMRI.

**Consent:** The authors confirm that written consent for submission and publication of this case report including the images and associated text has been obtained from the patient in line with the Committee on Publication Ethics (COPE) guidance.

**Funding:** None declared.
